# Supplementary material for: Point estimation for adaptive trial designs II: practical considerations and guidance
Source: Stat Med. Author manuscript; Available in PMC 2023 Jun 30. (PMC7614609; doi:10.1002/sim.9734)
Supplement: Supplementary file 1 [file EMS173406-supplement-Supplementary_Material.pdf]

Appendix

A.1 Guidance on bias-adjusted analyses for adaptive designs

FDA: Adaptive Designs for Clinical Trials of Drugs and Biologics

“Adaptive designs require specific analytical methods to avoid increasing the chance of erroneous conclusions and introducing bias in estimates. For complex adaptive designs, such methods may not be readily available, and simulations are often critical” - page 6

“It is important that clinical trials produce sufficiently reliable treatment effect estimates to facilitate an evaluation of benefit-risk and to appropriately label new drugs, enabling the practice of evidence-based medicine. Some adaptive design features can lead to statistical bias in the estimation of treatment effects and related quantities. For example, each of the two cases of Type I error probability inflation mentioned in section III.A. above has a potential for biased estimates. Specifically, a conventional end-of-trial treatment effect estimate such as a sample mean that does not take the adaptations into account would tend to overestimate the true population treatment effect. This is true not only for the primary endpoint which formed the basis of the adaptations, but also for secondary endpoints correlated with the primary endpoint. Furthermore, confidence intervals for the primary and secondary endpoints may not have correct coverage probabilities for the true treatment effects.

For some designs there are known methods for adjusting estimates to reduce or remove bias associated with adaptations and to improve performance on measures such as the mean squared error (e.g., Jennison and Turnbull 1999; Wassmer and Brannath 2016). Such methods should be prospectively planned and used for reporting results when they are available. Biased estimation in adaptive design is currently a less well-studied phenomenon than Type I error probability inflation, however, and methods may not be available for other designs. For these other designs, the extent of bias in estimates should be evaluated, and treatment effect estimates and associated confidence intervals should be presented with appropriate cautions

regarding their interpretation.” - page 8

“Finally, conventional fixed sample estimates of the treatment effect such as the sample mean tend to be biased toward greater effects than the true value when a group sequential design is used. Similarly, confidence intervals do not have the desired nominal coverage probabilities. Therefore, a variety of methods exist to compute estimates and confidence intervals that appropriately adjust for the group sequential stopping rules (Jennison and Turnbull 1999). To ensure the scientific and statistical credibility of trial results and facilitate important benefit-risk considerations, an approach for calculating estimates and confidence intervals that appropriately accounts for the group sequential design should be prospectively planned and used for reporting results.” - pages 12-13

“Consider group sequential designs: It is widely understood that multiple analyses of the primary endpoint can inflate the Type I error probability and lead to biased estimation of treatment effects on that endpoint. Less well appreciated, however, is that Type I error probability inflation and biased estimation can also apply to any endpoint correlated with the primary endpoint (Hung et al. 2007).” - page 22

[Documentation Prior to Conducting an Adaptive Trial] “Evaluation and discussion of the design operating characteristics, which should typically include Type I error probability; power; expected, minimum, and maximum sample size; bias of treatment effect estimates; and coverage of confidence intervals. Such evaluations might be achieved through analytical calculations and/or computer simulations. If operating characteristics are evaluated analytically, appropriate details (e.g., literature references or proofs) for the methodology should be submitted.” - page 28

“Appropriate reporting of the adaptive design and trial results … For example, the trial summary should describe the adaptive design utilized. In addition, treatment effect estimates should adequately take the design into account, or if naive estimates such as unadjusted sample means are used, the extent of bias should be evaluated, and estimates should be presented with appropriate cautions regarding their interpretation.” - page 30

The Adaptive designs CONSORT Extension (ACE) statement

“A goal of every trial is to provide reliable estimates of the treatment effect for assessing benefits and risks to reach correct conclusions. Several statistical issues may arise when using an AD depending on its type and the scope of adaptations, the adaptive decision-making criteria and whether frequentist or Bayesian methods are used to design and analyse the trial.

Conventional estimates of treatment effect based on fixed design methods may be unreliable when applied to ADs (for example, may exaggerate the patient benefit). Precision around the estimated treatment effects may be incorrect (for example, the width of confidence intervals may be incorrect). Other methods available to summarise the level of evidence in hypothesis testing (for example, p-values) may give different answers. Some factors and conditions that influence the magnitude of estimation bias have been investigated and there are circumstances when it may not be of concern. Secondary analyses (for example, health economic evaluation) may also be affected if appropriate adjustments are not made. Cameron et al. discuss methodological challenges in performing network meta-analysis when combining evidence from randomised trials with ADs and fixed designs. Statistical methods for estimating the treatment effect and its precision exist for some ADs and implementation tools are being developed. However, these methods are rarely used or reported and the implications are unclear. Debate and research on inference for some ADs with complex adaptations is ongoing. In addition to statistical methods for comparing outcomes between groups (item 12a), we specifically encourage authors to clearly describe statistical methods used to estimate measures of treatment effects with associated uncertainty (for example, confidence or credible intervals) and p-value (when appropriate); referencing relevant literature is sufficient. When conventional or naïve estimators derived from fixed design methods are used, it should be clearly stated. In situations where statistical simulations were used to either explore the extent of bias in estimation of the treatment effects … or operating characteristics, it is good practice to mention this and provide supporting evidence (item 24c)." - page 16

"For AD randomised trials, further discussion should include the implications of: …
- Potential bias and imprecision of the treatment effects if naïve estimation methods were used;" - page 21

"For some AD randomised trials, methods to derive statistical properties analytically may not be available. Thus, it becomes necessary to perform simulations under a wide range of plausible scenarios to investigate the operating characteristics of the design (item 7a), impact on estimation bias (item 12b), and appropriateness and consequences of decision-making criteria and rules. In such cases, we encourage authors to reference accessible material used for this purpose (for example, simulation protocol and report, or published related material). Furthermore, it is good scientific practice to reference software, programs or code used for this task to facilitate reproducible research." - page 24

A.2 Case study: Group sequential design

Definition of the information at stages 1 and 2

At stage k (k = $1,2$), let $\tilde{p}_k$ denote the pooled estimate of the mean overall success probability, i.e., the total number of observed successes divided by the total number of subjects. Then the observed information I_k is given by

$$I_k = \frac{1}{\tilde{p}_k(1-\tilde{p}_k)(1/n_{0k}+1/n_{CEk})}$$

where n_{0k} and n_{CEk} are the number of subjects on the placebo and CE arms, respectively, at stage k.

Definition of the conditional density of $\hat{\theta}$

The conditional density of $\hat{\theta}$, conditional on continuing to stage 2, is given by the following expression:

$$f(\hat{\theta}|T=2) = \frac{1-\Phi(\frac{c/\sqrt{I_1}-\hat{\theta}}{1/I_1-1/I_2})}{1-\Phi(c-\theta\sqrt{I_1})}\frac{\exp[-\frac{I_2}{2}(\hat{\theta}-\theta)^2]}{\sqrt{2\pi/I_2}}$$

where c is the stopping boundary at stage 1, i.e. the trial stops at stage 1 if $Z_1 \geq c$.

Conditional versus unconditional perspectives

Below we give some quotations from the literature focusing on the issue of the conditional versus unconditional perspective in the context of group sequential designs.

Troendle and Yu (1999)[100(pp1617-1618)]:

"Suppose a group sequential clinical trial is undertaken to determine the effect of an experimental drug on the state of a certain disease. Now suppose it is known that the trial was stopped at the first interim analysis because of treatment efficacy, and that the estimated treatment effect was X_1 - Y_1, the difference in sample means from the two groups… Is X_1 - Y_1 a reasonable estimate of the effect size? Although X_1 - Y_1 is unbiased, the general estimator X_T - Y_T, where T is the random stopping time, is known to be biased. Recently, an unbiased estimator ... and an essentially unbiased estimator ... have been developed for this problem. However, as will be shown later, these methods remain unbiased by overestimating

the effect when there is early stopping while underestimating the effect when the trial stops later. The overall effect is an unbiased estimator, but does that leave the scientist, who knows $T = 1$ any happier? We propose conditioning on the stopping time in a group sequential trial to reduce the discrepancy between the conditional expectation of the estimator and the parameter value."

Fan et al. (2004)[101(pp506-507)]:

"We also note that the bias referred to is the marginal or overall bias [i.e. the unconditional bias]. As much as the importance of the marginal bias, sometimes we will also face the question of what the potential bias is given the fact that the study is already stopped at this time, especially when it is a very early interim stage. To answer this question, we feel it is more relevant to investigate the bias conditioning on the actual stopping time. In this paper, we focus on the angle of the conditional bias and in the meanwhile also keep in mind the marginal bias."

"The conditional method is not meant to replace the unconditional methods because these two methods are developed to address different issues. Instead it is proposed as an addition and alternative means that we can take advantage of when the conditional bias is more concerning, rather than a replacement to the unconditional methods."

Zhang et al. (2012)[102(p4876)]:

"Although this article focuses on the bias conditional on the observed stopping time, we also recognize the importance of the marginal or unconditional bias … Evaluation of the unconditional bias is particularly helpful in the trial design stage; however, there is also value in assessing the potential bias given that the trial has already stopped (conditional bias), especially on the basis of a very early interim analysis. Fan and colleagues found that the conditional bias may be quite serious, even in situations in which the unconditional bias is acceptable[101]. Most of the available adjustment methods focus on the unconditional bias, which has little effect on the conditional bias.

Schoenbrot and Wagenmakers (2018)[103(p140)]:

"Although sequential designs have negligible unconditional bias, it may nevertheless be desirable to provide a principled "correction" for the conditional bias at early terminations, in particular when the effect size of a single study is evaluated."

Shimura et al. (2017)[104(p2068)]:

"A reduction in conditional bias is as important as a reduction in overall bias because, in practice, researchers can only obtain an estimate that is conditional on the stopping stages."
